# Supplementary material for: Splice-Junction-Based Mapping of Alternative Isoforms in the Human Proteome
Source: Cell Rep. Author manuscript; Available in PMC 2020 Jan 15. (PMC6961840; doi:10.1016/j.celrep.2019.11.026)
Supplement: 2 [file NIHMS1546469-supplement-2.zip › DF1/PXD006675/InferiorVenaCava/InferiorVenaCava_6_RALGDS_SAGPAGGAEPLFPGSR.pdf]

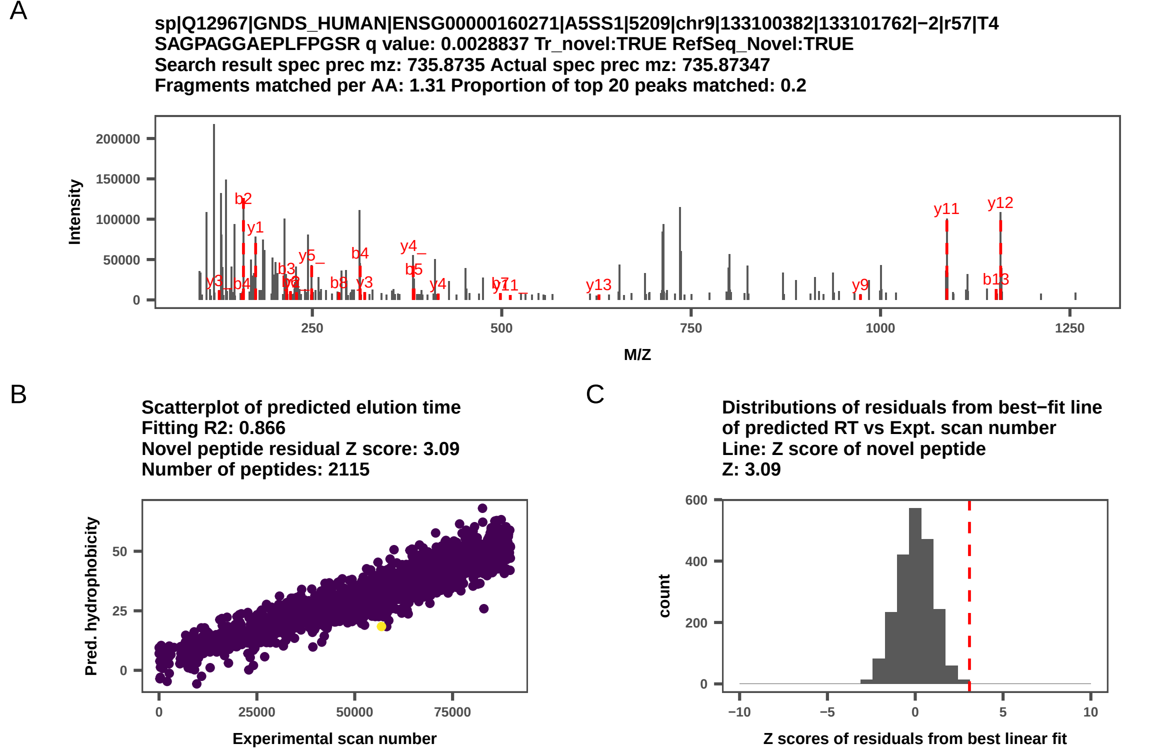
A
sp|Q12967|GNDS_HUMAN|ENSG00000160271|A5SS1|5209|chr9|133100382|133101762|−2|r57|T4
SAGPAGGAEPLFPGSR q value: 0.0028837 Tr_novel:TRUE RefSeq_Novel:TRUE
Search result spec prec mz: 735.8735 Actual spec prec mz: 735.87347
Fragments matched per AA: 1.31 Proportion of top 20 peaks matched: 0.2
Intensity
200000
150000
100000
50000
0
250
500
750
1000
1250
M/Z
y11
y12
b13
y13
y9
B
Scatterplot of predicted elution time
Fitting R2: 0.866
Novel peptide residual Z score: 3.09
Number of peptides: 2115
Pred. hydrophobicity
50
25
0
25000
50000
75000
Experimental scan number
C
Distributions of residuals from best−fit line
of predicted RT vs Expt. scan number
Line: Z score of novel peptide
Z: 3.09
count
600
400
200
0
−10
−5
0
5
10
Z scores of residuals from best linear fit
